# Anti-Proliferative Effect of *Rosmarinus officinalis* L. Extract on Human Melanoma A375 Cells

**DOI:** 10.1371/journal.pone.0132439

**Published:** 2015-07-15

**Authors:** Lucia Cattaneo, Rosella Cicconi, Giuseppina Mignogna, Alessandra Giorgi, Maurizio Mattei, Giulia Graziani, Rosalia Ferracane, Alessandro Grosso, Patrizia Aducci, M. Eugenia Schininà, Mauro Marra

**Affiliations:** 1 Department of Biology, University of “Tor Vergata”, Rome, Italy; 2 Centro di Servizi Interdipartimentale, Stazione per la Tecnologia Animale, University of “Tor Vergata”, Rome, Italy; 3 Department of Biochemical Sciences, Sapienza, University of Rome, Rome, Italy; 4 Department of Agricultural and Food Science, University of Naples “Federico II”, Portici, Naples, Italy; Universidad de Malaga, SPAIN

## Abstract

Rosemary (*Rosmarinus officinalis* L.) has been used since ancient times in traditional medicine, while nowadays various rosemary formulations are increasingly exploited by alternative medicine to cure or prevent a wide range of health disorders. Rosemary’s bioproperties have prompted scientific investigation, which allowed us to ascertain antioxidant, anti-inflammatory, cytostatic, and cytotoxic activities of crude extracts or of pure components. Although there is a growing body of experimental work, information about rosemary’s anticancer properties, such as chemoprotective or anti-proliferative effects on cancer cells, is very poor, especially concerning the mechanism of action. Melanoma is a skin tumor whose diffusion is rapidly increasing in the world and whose malignancy is reinforced by its high resistance to cytotoxic agents; hence the availability of new cytotoxic drugs would be very helpful to improve melanoma prognosis. Here we report on the effect of a rosemary hydroalcoholic extract on the viability of the human melanoma A375 cell line. Main components of rosemary extract were identified by liquid chromatography coupled to tandem mass spectrometry (LC/ESI-MS/MS) and the effect of the crude extract or of pure components on the proliferation of cancer cells was tested by MTT and Trypan blue assays. The effect on cell cycle was investigated by using flow cytometry, and the alteration of the cellular redox state was evaluated by intracellular ROS levels and protein carbonylation analysis. Furthermore, in order to get information about the molecular mechanisms of cytotoxicity, a comparative proteomic investigation was performed.

## Introduction

Rosemary (*Rosmarinus officinalis* L.), is a shrub of the Lamiaceace family, which has been widely used in the Mediterranean area since ancient times, both as a culinary spice, in order to preserve food or improve its taste, as well as in folk medicine, as a medicinal herb. Nowadays rosemary extracts, in various formulations, are increasingly exploited by the alternative medicine or by the functional food industry, due to their benefits on human health. In fact, traditional medicine used rosemary for centuries to cure various disorders, *i*.*e*. as antispasmodic in renal colic or dysmenorrhea, as a relief for respiratory disorders and as smooth muscle relaxant [1]. In more recent years, a growing body of experimental investigation confirmed the pharmacological potential of rosemary, expanding also the range of its possible therapeutic applications. In fact, administration of crude extracts to mice, rats or rabbits demonstrated chemoprotective effects against hepatotoxicity [2] or cirrhosis [3], against arterial thrombotic diseases [4], gastric ulcerative lesions [5], hyperglycaemia [6], neurogenic and inflammatory pain [7], as well as antibacterial and antioxidant activities [8,9]. On the other hand, modern techniques of analysis have led to the isolation and identification of many of the components of rosemary extracts, thereby prompting studies on the pharmacological effects of pure bioactive principles and allowing the investigation of the underlying molecular mechanisms. The phenolic diterpenes carnosol and carnosic acid and the phenolic acid rosmarinic acid are the main antioxidant components, while a number of flavonoids are usually also present, among which scutellarin, luteolin, genkwanin and kaempferol are major constituents [9,10]. A growing body of evidence indicates that rosemary exerts anticancer effects on various *in vitro* and *in vivo* model systems and that different cellular mechanisms may be involved. Rosemary extracts exhibited anti-proliferative activity in a number of different tumor cell lines [9,11,12], a property which has been correlated to the occurrence of specific compounds, particularly carnosol, carnosic acid, rosmarinic acid [13] and partially to their antioxidant power [9]. Rosmarinic acid induced apoptosis in colorectal cancer cells [14], possibly interfering with the MAPK/ERK pathway. Rosemary essential oil down-regulated the expression of the bcl-2 gene, while up-regulated that of the bax gene, in liver cancer cells [15]. Carnosic acid inhibited proliferation of human myeloid leukemia cells without inducing apoptosis [16]. Carnosol inhibited the migration of metastatic mouse melanoma B16/F10 cells *in vitro* by suppressing the expression of metalloproteinase-9 [17]. Rosemary antitumor activity was evidenced also *in vivo*: survival of rats with acute myeloid leukemia was increased by administration of rosemary crude extracts or carnosol, in combination with 1α-25 dihydroxy vitamin D (3), which determined a strong antiproliferative effect [18,19]. Carnosol was able to reduce tumor molteplicity in a mouse model of colonic tumorigenesis [20]. *In vitro* and *in vivo* data indicated also that rosemary crude extracts or purified components exerted chemoprotective effects, by inhibiting early phases of tumor development, or contrasting the effect of chemical mutagenic compounds [21,22,23,24]. Mechanisms of chemoprotection probably involved inhibition of phase I enzymes of carcinogenesis, as well as increased expression of detoxifying enzymes [25]. Melanoma is a skin malignant tumor induced by transformation of melanocytes [26], whose incidence rate is rapidly increasing in the world [27]. Metastatic melanoma has a very poor prognosis, a fact that is in part due to its high resistance to cytotoxic agents [28,29]. Hence, finding new sources of anti-cancer compounds, to improve melanoma prognosis is a relevant research issue and plant extracts, containing many constituents with diverse and synergistic biological effects may greatly contribute to integrate or enhance chemical treatments. In the present paper we present data about the effect of a *Rosmarinus officinals* L. hydroalcoholic extract, on the viability of the human melanoma A375 cell line. Main components of rosemary extract were identified by liquid chromatography coupled to tandem mass spectrometry (LC/ESI-MS/MS) and the effect of crude extract or of pure components on the proliferation of cancer cells was tested by MTT and Trypan blue assays. The effect on cell cycle was investigated by flow cytometry and alteration of the cellular redox state evaluated by intracellular ROS production determination and protein carbonylation analysis. Finally, to obtain hints about underlying molecular mechanisms, a comparative proteomic analysis was performed.

## Materials and Methods

### Preparation of *Rosmarinus officinalis* extract

The hydroalcoholic extract of *Rosmarinus officinalis* was kindly supplied by the phytochemical company Sarandrea Marco & Co. S.r.l., Collepardo (FR), Italy. The extract was made using leaves of *Rosmarinus officinalis* specimens endemic of the Ciociaria area in the Lazio region, in central Italy. Leaves were harvested from plants in the vegetative stage and, within 24 hours of collection, ground into fine powder and suspended at 330g/L in a solution of 65% (w/w) ethanol/water, for 21 days at room temperature. The extract was then filtered and stored to -20°C until use.

### Liquid chromatography

Chromatographic separation was performed using an HPLC apparatus equipped with two Micropumps Series 200 (PerkinElmer, Shellton, CT, USA) and a Prodigy ODS3 100 Å column (250 mm × 4.6 mm, particle size 5 μm) (Phenomenex, CA, USA). The eluents were: solvent A: water containing 0.2% formic acid; solvent B: CH_3_CN/CH_3_OH (60:40, v/v). The gradient program was as follows: 50% B (0 min), 100% B (15 min), 100% B (15–35 min), 50% B (40 min), 50% B (40–45 min) at a constant flow of 0.8 mL/min. The LC flow was split and 0.2 mL/min was sent to the mass spectrometry. Three injections were performed for each sample. Injection volume was 20 μL. Mass spectrometry analyses of extracts were performed on an API 3000 triple quadrupole mass spectrometer (Applied Biosystems, Canada) equipped with a TurboIonSpray source, working both in the negative and positive ion mode. The analyses were performed using the following settings: drying gas (air) was heated to 400°C, capillary voltage (IS) was set to 4000 V and 5000 V in negative and positive ion mode, respectively.

Quantitative HPLC analysis of main components was performed on a LC-20 Prominence HPLC system (Shimadzu, Japan), equipped with a LC-20AT quaternary gradient pump, a SPD-M20A photo diode array detector (PDAD) and a SIL-20 AH autosampler or a Rheodyne 7725i valve, with a 20 μLfixed loop. The extract was separated on a Phenomenex Kinetex C_18_ column (2.6 μm, 100 x 4.60 mm; Phenomenex, CA, USA). The mobile phase consisted of: solvent A: water containing 0.2% (v/v) TFA; solvent B: CH_3_CN/CH_3_OH (60:40, v/v). A binary gradient was used for elution: 15% B (0 min), 35% B (3 min), 75% B (9 min), 15% B (11–15 min). The mobile phase flow rate was 0.8 mL/min; spectra were recorded between 190–400 nm. Column temperature was controlled at 40°C. Separated compounds were identified by comparison of their retention times and UV spectra with those of the following authentic standards: apigenin (A3145 Sigma), luteolin (72511, Sigma), caffeic acid (CO265, Sigma), scutellarin (73577, Sigma), carnosol (C9617, Sigma), rosmarinic acid (00390580, Sigma), respectively. These compounds were also used to build up calibration curves in the range 5 to 500 μg/mL. For quantitative analysis different concentrations of unknown samples were injected in triplicate. Reported values represent the means ± SD of three independent extractions.

### Cell culture

Human melanoma A375 cells (ATCC; Manassas, VA, USA) or B16-F10 murine melanoma cells (ATCC; Manassas, VA, USA) were cultured in RPMI-1640 medium, supplemented with 10% (v/v) fetal bovine serum (FBS), 1% L-glutamine (v/v), 100 units/mL penicillin and 100 μg/mL streptomycin. The cells were grown at 37°C with 5% CO_2_ in a humidified atmosphere.

### Determination of cell viability

Cell viability was assessed by MTT [3-(4,5-dimethylthiazol-2-yl)-2,5-diphenyl tetrazolium bromide] and Trypan blue assays. For MTT assay, 2x10^3^ cell/well were seeded into sterile 96-well plates and incubated overnight. The day after, cells were treated with increasing concentrations of rosemary extract, or of main pure components, namely luteolin, carnosol, scutellarin, rosmarinic acid and apigenin, and incubated for 24, 48 and 72 h, respectively. After incubation, 0.5μg/μL MTT (Sigma) was added and cells incubated for additional 4 h at 37°C in the dark. Then, the medium was removed and formazan crystals were dissolved in DMSO and cellular metabolism was determined by monitoring the color development at 570 nm, in a multi-well scanning spectrophotometer (Sunrise, Tecan, CH). IC_50_ values were estimated following 72 h incubation. For Trypan blue assay, A375 cells were seeded at a density of 2 x10^4^ cells/well in sterile 24-well plates. After 24 h, cells were treated with increasing concentrations of rosemary extract and incubated for 24, 48 and 72 h. Then, adherent cells were washed, detached with trypsin 0.05% (w/v), EDTA 0.02% (w/v), (Sigma), stained with 0.4% Trypan blue (w/v), (Sigma) and counted in triplicate in an optic microscope, to estimate the number of live cells. Cell viability was expressed as a percentage of live treated cells with respect to live control cells.

### Flow cytometry analysis of cell cycle

2 x10^4^ cell/well were seeded into sterile 24-well plates and after 24 h, rosemary extract at 1: 120 and 1:240 dilution, or main components of the rosemary extract at a concentration of 20 μM, were added to the cell culture medium. Cells were also treated with ethanol, as vehicle control. After incubation for 24, 48 and 72 h, cells were detached with trypsin-EDTA, washed with PBS, collected by centrifugation at 450 x g for 10 min and stained with propidium iodide (PI) staining solution (Sigma), containing 50 μg/mL PI (w/v), 0.5% RNase A (w/v) (Sigma) and 0.1% Triton-X 100 (v/v), After incubation for 30 min at 4°C in the dark, cell cycle distribution was analyzed by flow cytometry on a FACS Calibur flow cytometer (Becton-Dickinson, Mountain View, CA). A total of 10,000 events in each sample was acquired. Cells cycle distribution was determined by using the Cellquest Pro software (Becton-Dickinson).

### Determination of intracellular reactive oxygen species (ROS)

Intracellular ROS generation was measured by using the fluorescent probe 5-(and 6)-chloromethyl-20,70-dichlorodihydrofluorescein diacetate, acetyl ester (CM-H2DCFDA, Molecular Probes/Invitrogen; Carlsbad, CA, USA). 3 x10^5^ cell/well were seeded into sterile 24-well plates. After 24 h, rosemary extract was added to the cell culture medium at 1:120 and 1:240 dilutions. After incubation at 37°C in 5% CO_2,_ for 24 h, cells were detached with trypsin and incubated with 5 μM CM-H2DCFDA, in the dark, at 37°C. After 30 minutes of incubation, cells were centrifuged and the pellet was washed twice with ice-cold PBS. The pellet was then resuspended in FACS buffer (0.5% BSA (w/v), 0.1% sodium azide (w/v) in PBS) and radical formation assessed by flow cytometry, in a FACS Calibur flow cytometer (Becton-Dickinson, Mountain View, CA). CM-H2DCFDA mean fluorescence was measured in FL-1 with an excitation wavelength of 488 nm and an emission wavelength of 530 nm. 10,000 events were evaluated for each analysis.

### Analysis of protein carbonylation

Carbonyl groups in side chains of proteins were detected using the OxyBlot protein oxidation detection kit (Millipore). Cells were cultured as above reported and treated with rosemary extract (1:120 and 1:240 dilutions) for 24h. After incubation, cells were detached with trypsin, washed twice with ice-cold PBS and centrifuged. The pellet was resuspended and incubated in Lysis Buffer (50 mM Tris-HCl pH 7.4, 1% Triton-X-100 (v/v), 250 mM NaCl, 5 mM EDTA) at 4°C, overnight. Cell lysates were centrifuged at 14,000 x g for 20 min and protein concentration in the supernatant determined by Bradford assay. Proteins were derivatized to 2,4-dinitrophenylhydrazone by 2,4-dinitrophenylhydrazine (DNPH), according to the manufacturer’s instructions, separated by SDS-PAGE and subjected to Western Blot. Oxidized proteins were detected by anti-2,4-dinitrophenylhydrazone antibodies.

### Two-dimensional gel electrophoresis (2D-E)

Sample preparation: cells were cultured as above reported and treated with rosemary extract (1:240) for 24h. After incubation, cells were detached with trypsin, washed twice with ice-cold PBS and centrifuged. Cell pellet was resuspend in Lysis Buffer (50 mM Tris-HCl pH 7.4, 1% Triton-X-100 (v/v), 250 mM NaCl, 5 mM EDTA) and incubated overnight at 4°C. Cell lysates were centrifuged at 14,000 x g for 20 min and protein concentration in the supernatant was determined by Bradford assay. Equivalent protein amounts (300 μg) of control and treated cell samples were desalted by precipitation with cold ethanol (overnight at -20°C). Precipitates were centrifuged at 15,000 x g for 15 min and pellets solubilised in 200 μL of rehydration buffer (5 M urea, 2 M thiourea, 50 mM DTT, 2% (w/v) CHAPS, 0.2% (v/v), ampholytes pH 3–10). For first-dimension electrophoresis, sample solutions were loaded onto non linear pH 3–10 IPG ReadyStrips (Bio-Rad Laboratories S.r.l., Segrate, Milano, Italy). After passive rehydration for 12 h at 20°C, IEF was performed in a Protean IEF Cell Apparatus (Bio-Rad) as follows: (i) 250 V for 15 min; (ii) 250–8000 V in 2.5 h; (iii) 8000V for 5 h. Before second-dimension electrophoresis, strips were equilibrated for 15 min in 50 mM Tris-HCl (pH 6.8) containing 6 M urea, 2% (w/v) sodium dodecilsulfate (SDS), 20% (v/v) glycerol and 130 mM dithiothreitol, and then for other 15 min in the same buffer, containing 135 mM iodoacetamide, in place of dithiothreitol. The second dimension was carried out on pre-casted 4–12% Bis-Tris gels, by using a Criterion apparatus (Bio-Rad) and MES-SDS solution as running buffer (Bio-Rad), applying a ramping voltage (from 100 to 200 V). The 2-D gels were stained with colloidal Coomassie. After destaining, gels were digitalized using a computing densitometer (GS-710 Imaging Densitometer; Bio-Rad). The images were analyzed for the detection, matching and quantification of protein spots, using PD Quest software [version 8.0.1 (Bio-Rad)], according to the manufacturer’s procedures. Manual inspection of the spots was performed to verify the accuracy of automated gel matching; any errors in the automatic procedure were corrected prior to quantitative analysis. After normalization of the spot densities against the whole gel densities, the percentage volume of each spot was averaged for nine gels (three replicates of three different biological samples) and compared between groups (control and treated with the rosemary extract) to find out statistically significant (Student’s t-test, P ≤ 0.05) differences. A two-fold change in normalized spot densities was considered indicative of a differentially expressed component.

### Protein identification by mass spectrometry

Selected spots were manually excised and subjected to in-gel trypsin proteolysis. Briefly, after destaining steps, using 50 mM NH_4_HCO_3_ (15 min), 50% CH_3_CN in 50 mM NH_4_HCO_3_ (v/v), (10 min) and 100% CH_3_CN (15 min), 100 ng of trypsin (Trypsin Gold, Mass Spectrometry Grade, Promega, Madison, WI, USA), solubilized in 10 μL of 25 mM NH_4_HCO_3_ digestion buffer, were added to vacuum-dried gel. Digestion was performed at 37°C overnight. An aliquot of each peptide mixture was mixed with the same volume of CHCA matrix solution (5 mg/mL) in 70% CH_3_CN, containing 0.1% TFA (v/v) and spotted onto an appropriate MALDI target plate. MALDI-ToF MS analyses were performed with an AutoFlex II instrument (Bruker Daltonics, Bremen, Germany), equipped with a 337 nm nitrogen laser and operating in reflector positive mode. Two tryptic autolytic peptides were used for the internal calibration (*m/z*842.5100 and 2211.1046). Data were analyzed by flex Analysis program (Bruker Daltonics, Bremen, Germany). Identification by peptide mass fingerprint (PMF), with the mono-isotopic mass list, was performed using BioTools program (Bruker Daltonics, Bremen, Germany), by the Mascot search engine, against human SwissProt database [(SwissProt 2014_01 (542258 sequences; 192776118 residues)]. Up to two missed cleavage, 50 ppm measurement tolerance, oxidation at methionine (variable modification) and carbamidomethylation at cysteine (fixed modification) were considered. Identifications were validated when the probability-based Mowse proteins core was significant according to Mascot [30].

### Western blot analysis

Protein extracts (approximately, 15 μg) were resolved on 4–15% Mini-PROTEAN TGX Precasted gels by sodium dodecyl sulfate (SDS)-polyacrylamide gel electrophoresis (SDS-PAGE; 200 V, 45 min). The protein bands were electrotransferred to nitrocellulose membranes (80 V, 120 min). Membranes were then treated with a 5% enhanced chemiluminescence (ECL) blocking agent (GE Healthcare Bio-Sciences) in a saline buffer (T-TBS) containing 0.1% Tween-20, 10 mM Tris-HCl, 150 mM NaCl, 1 mM CaCl_2_, and 1 mM MgCl2, pH 7.4, for 1 h and then incubated with the primary antibody overnight at 4°C. Subsequently, membranes were washed three times in T-TBS, and the bound antibodies were detected using appropriate horseradish peroxidase-conjugated secondary antibodies, followed by an ECL Plus Western blotting detection system (GE Healthcare Bio-Sciences). ECL was detected using a Molecular Imagers ChemiDoc mod. MP System (Bio-Rad Laboratories), and acquired using ImageLab Software, ver. 4.1. Immunodetection was carried out using goat polyclonal antibodies (SantaCruz Biotechnology) against poly (rC)-binding protein 1 (PCB1; sc-16504, dilution 1:200), poly (rC)-binding protein 2 (PCB2; sc-30725, dilution 1:200), neutral alpha-glucosidase AB (GANAB; sc-20279, dilution 1:200), Lamin A (LMN A/C; sc-6215 dilution 1:200). The anti-PDIA3 antibody was gift by prof. Fabio Altieri (dilution 1:2000). In each analysed sample, the signal of the target protein was normalized to the corresponding Glyceraldehyde 3-phosphate dehydrogenase (Santa Cruz Biotechnology, GAPDH; sc-32233 dilution 1:500). Three replicates were performed, one for each biological sample. All results are expressed as mean ± SD. Differences between experimental groups were determined by Student’s t-test. The P-value of ≤ 0.05 was considered statistically significant.

## Results and Discussion

### Identification and quantification of rosemary extract components

**Fig 1:**
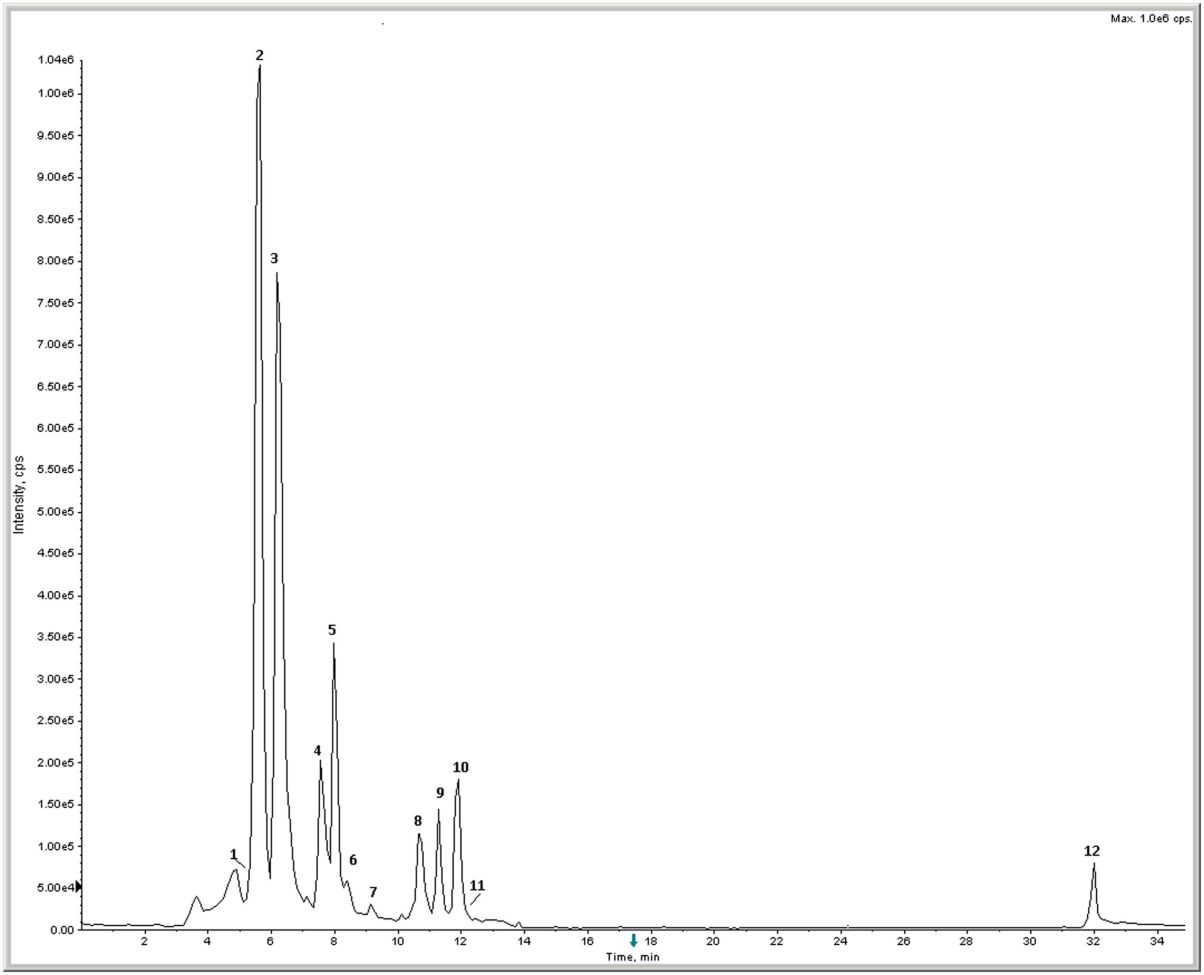
Total Ion Chromatogram (TIC) of rosemary extract. Numbers refer to compounds listed in Table 1.

Principal components of the rosemary extract were successfully identified using LC-MS/MS. The MS chromatogram is shown in Fig 1. The compounds were identified interpreting their MS, MS/MS and UV spectra (not shown) and comparing their data with those reported in the literature [31,32,33,34]. The identified compounds are listed in Table 1, including retention times, molecular weight, MS/MS fragments, as well as their proposed identifications. The quantitative composition of the extract was carried out by HPLC with diode array detection and calibration curves with pure standards. As reported in Table 1, major components were: rosmarinic acid, luteolin, apigenin, carnosol, caffeic acid and scutellarin. On the overall, the profile of metabolites of the rosemary extract appears qualitatively in accordance with others reported in the literature [9,10,31,33].

**Table 1:** List of metabolites occurring in the *Rosmarinus officinalis* extract identified by LC MS/MS.

Peak	Retention Time (min)	m/z	+/-	Major fragments *(m/z)*	Compound	Concentration (μg/ml)
1	5.18–6.28	461	-	299/284	Homoplantaginin	n. d.
2	5.60	359	-	197/179/161	Rosmarinic acid	398.1
3	6.28	461	-	285	Scutellarin	23.6
4	7.65	285	-	151	Luteolin	199.5
5	7.74	285	-	267/241	Scutellarein	n. d.
6	8.52	179	-	135	Caffeic Acid	114.4
7	9.25	269	-	151	Apigenin	39.6
8	10.96	347	+	301/283	Rosmanol	n. d.
9	11.30	315	+	297/300/282	Cirsimaritin	n. d.
10	11.92	329	-	285/211	Carnosol	80.1
11	11.93	331	-	287	Carnosic Acid	n. d.
12	32.07	373	-	329/293	Rosmarinic acid methylester	n. d.

Concentrations were determined by means of calibration curves with pure standards, as reported in material and methods (n. d. = not determined).

### Effect of rosemary extract treatment on the viability of melanoma cells

**Fig 2:**
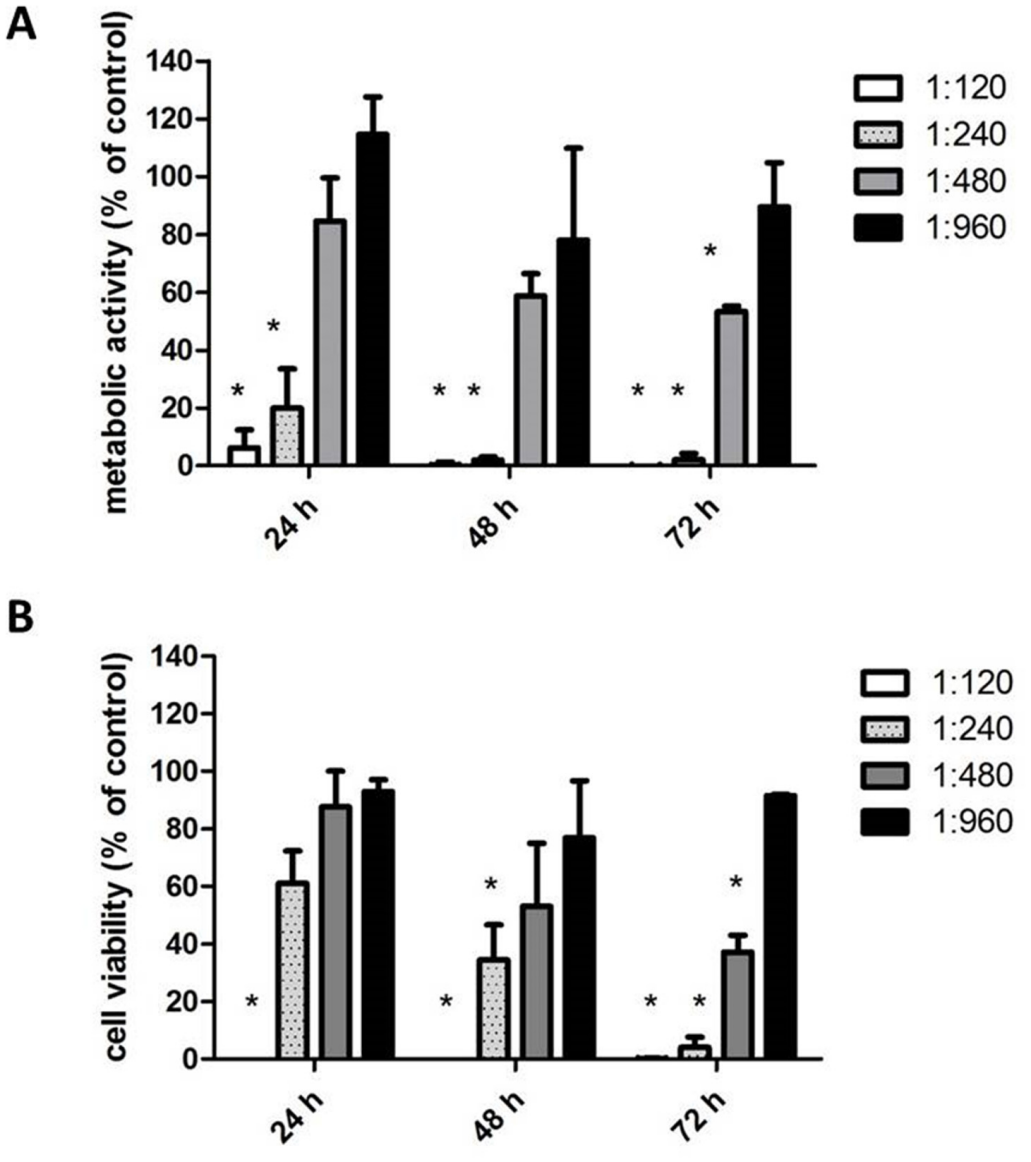
Effect of *Rosmarinus officinalis* extract on A375 melanoma cells. (A) Metabolic activity (MTT test). (B) Cell viability (Trypan blue exclusion test). Data are expressed as % of cell survival with respect to control. Results are the mean ± SD from three independent experiments. * P ≤ 0.05 versus vehicle control.

The effect of the hydroalcoholic extract of *Rosmarinus officinalis* on the viability of human melanoma A375 cell line (ATCC; Manassas, VA, USA), was assayed measuring the mitochondrial activity of living cells, by the MTT test. Results, reported in Fig 2 (panel A), showed that rosemary extract reduced cell growth in a time and dose-dependent manner. 1:120, 1:240 and 1:480 extract dilutions drastically reduced cellular metabolic activity. The anti-proliferative effect was evident already at 24 h and was enhanced at 48 and 72 h of incubation, whereas the 1:960 dilution was substantially ineffective at each time of incubation tested. The IC_50_ estimated after 72 h incubation, was 1:480.

MTT is an indirect colorimetric assay assessing metabolic activity, hence, in order to confirm the loss of survival rate through a more direct assay, a Trypan blue exclusion test was performed. Results, reported in Fig 2 (panel B), showed that the extract treatment brought about a time and dose-dependent reduction of melanoma cells proliferation, with a trend very similar to that observed in the MTT test. In fact, 1:120 and 1:240 dilutions, after 72 h incubation determined a drastic loss of cell proliferation, whereas the 1:960 dilution was ineffective. Furthermore, washing of treated cells, reseeding and culturing in the absence of the extract, did not result in recovery of growth (data not shown), indicating that the effect was irreversible, and therefore likely due to induction of differentiation processes. Similar results but at lower extract dilutions (1:60–1:240) were obtained on B16-F10 murine melanoma cells (S1 Fig). Hence on the overall, results suggested that treatment inhibited cell proliferation, consistently with previous studies demonstrating that rosemary extracts were able to inhibit growth of various tumor cells lines [9,11,35].

**Fig 3:**
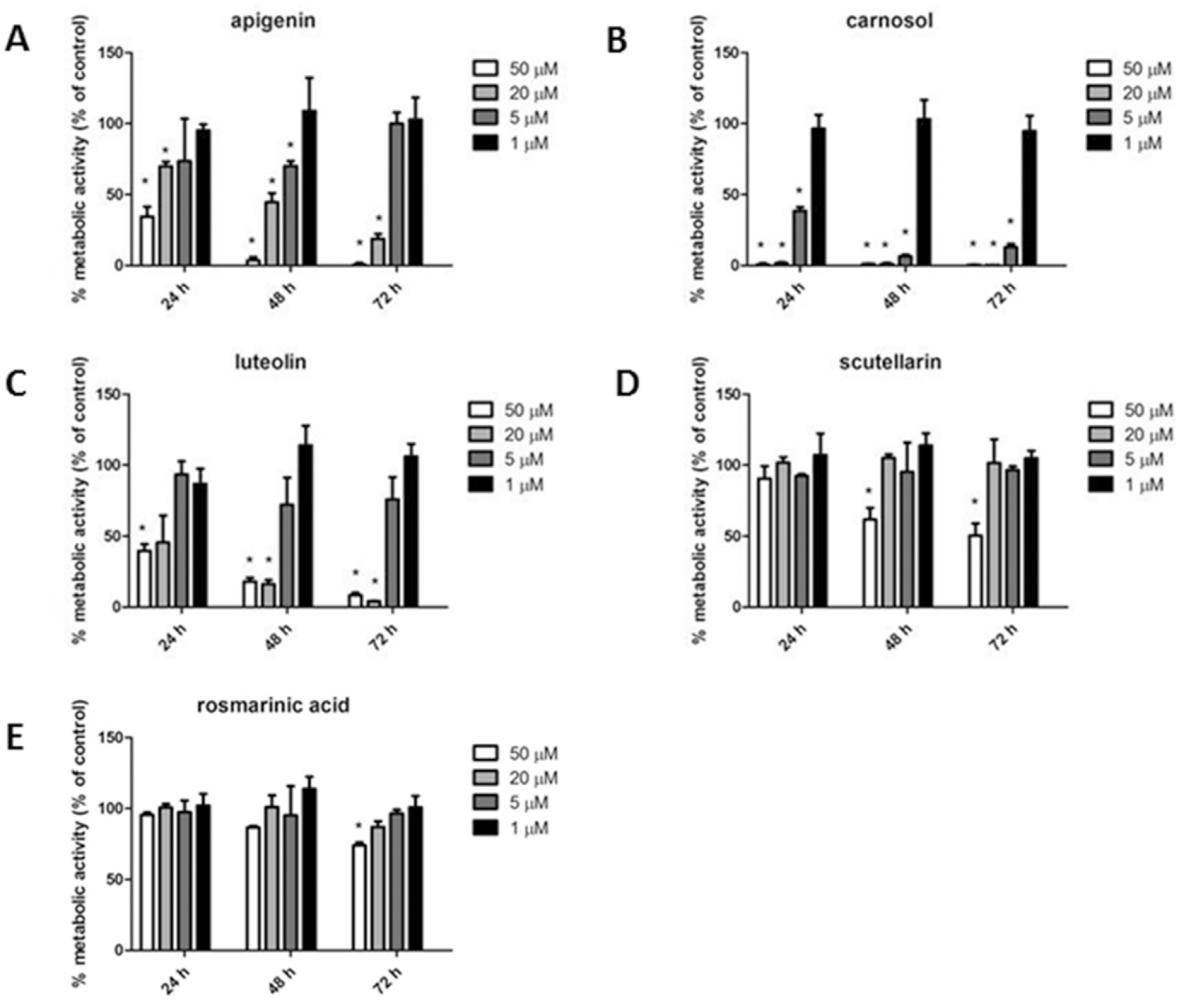
Effect of apigenin (A), carnosol (B), luteolin (C), scutellarin (D) and rosmarinic acid (E) on metabolic activity of A375 melanoma cells, assayed by MTT assay. Data are expressed as % of cell survival with respect to control. Results are the mean ± SD from three independent experiments. * P ≤ 0.05 versus vehicle control.

In order to ascertain to which substance(s) the antiproliferative activity could be ascribed, luteolin, carnosol, scutellarin, rosmarinic acid and apigenin [36,37], namely five major constituents of the rosemary extract (Table 1), were separately assayed by MTT test at 24, 48 and 72 h of incubation. Results, showed in Fig 3, indicated that, apigenin, luteolin and carnosol were much more effective than scutellarin and rosmarinic acid. These data are comparable to those from other authors, demonstrating a lower inhibitory activity for rosmarinic acid [12] and scutellarin [38] as compared to carnosol [39,40], luteolin [41,42,43] and apigenin [36,37,44]. However, since single substances resulted effective at concentrations (20, 50 μM) far exceeding those occurring in the rosemary extract, results suggested that cytotoxicity of the total extract resulted from the combination of different activities, possibly due to diverse molecules. In fact, indirect evidence exists that in herbal medicines multi-factorial effects can occur, which decrease the active concentration of pure components [45]. To test this possibility, the five pure compounds were tested in the MTT assay at the same concentrations occurring in the total extract (1: 120 dilution), as a reconstituted mixture. Under these conditions results were negative: the reconstituted mixture didn’t show any significant growth inhibitory activity (data not shown). A possible interpretation of this discrepancy is that additional compounds present in the total extract (as shown by HPLC-ms) significantly contribute to its overall cytotoxic activity, bringing about a network of combined effects more complex than that occurring in the reconstituted mixture.

### Effect of rosemary extract treatment on cell the cycle of melanoma cells

**Fig 4:**
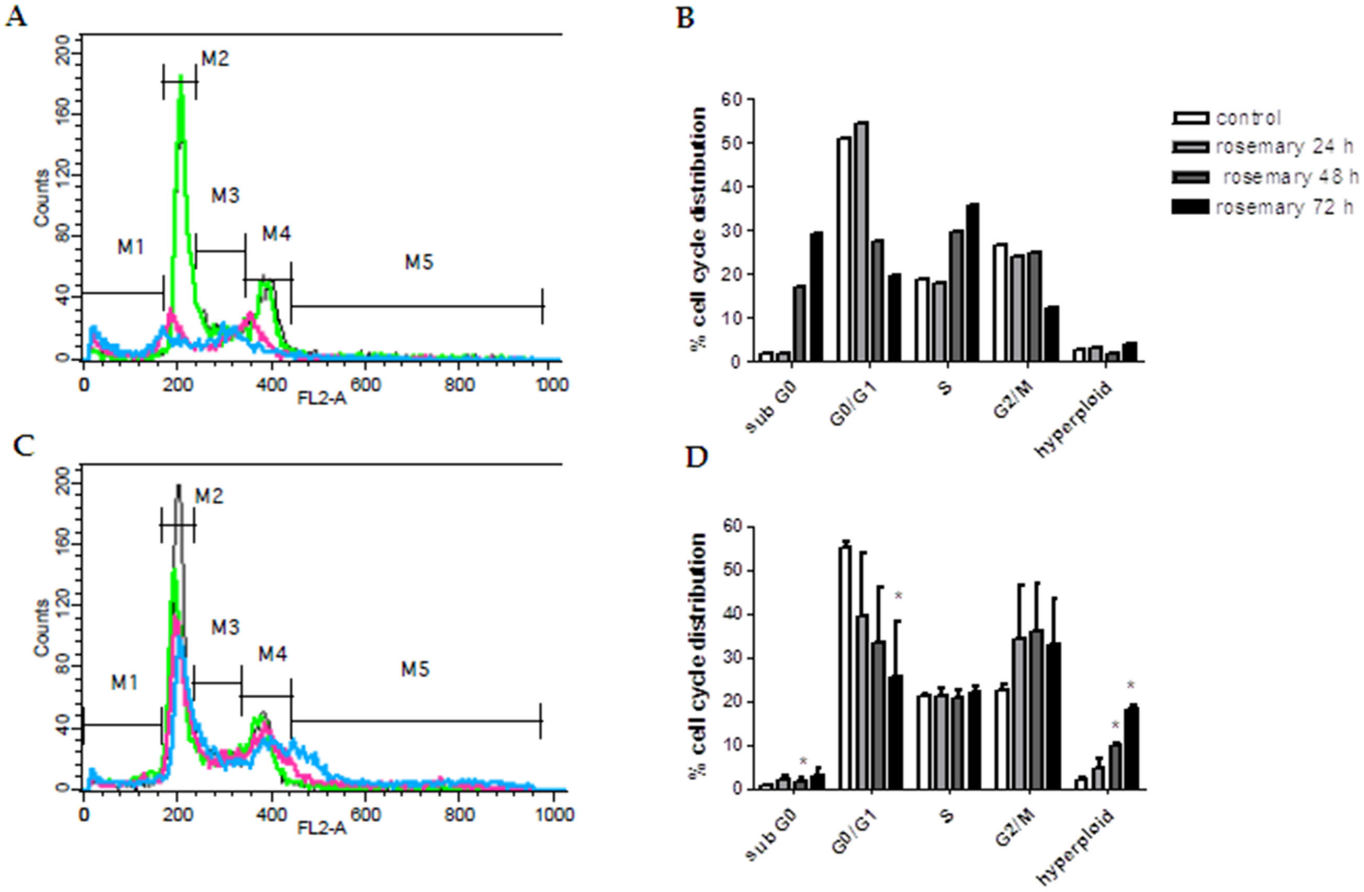
Cell cycle analysis of A375 melanoma cells treated with *Rosmarinus officinalis* extract. The figure shows the DNA content flow cytometric histograms of A375 cells (A-C) and the corresponding percentages of cell cycle distribution after treatment with *Rosmarinus officinalis* extract (B-D). Plots A and C show vehicle treated cells (black line) and cells treated for 24 (green line), 48 (fuchsia line) and 72 h (blue line) with 1:120 and 1:240 dilutions of *Rosmarinus officinalis* extract. M1, M2, M3, M4 and M5 are representative virtual markers of subG0/G1, G0/G1, S, G2/M and hyperploid phases of cell cycle, respectively. After treatment, cells were stained with propidium iodide and flow cytometric analysis was performed as described in Materials and Methods. The data shown in A and C are representative of three independent analyses. Results shown in B and D are the mean ± SD from three independent experiments. *P≤0.05 versus vehicle treated control cells.

The inhibition of cell viability could result from the induction of apoptosis and/or cell growth arrest, so, in order to get information about the cellular processes possibly affected by the rosemary extract, the effect on cell cycle was investigated by flow cytometry. To this purpose A375 melanoma cells were incubated with different dilutions of crude extract, for 24, 48 and 72 h, then labelled with propidium iodide and subjected to FACS analysis. Results reported in Fig 4, showed that treatments at 1:120 and 1:240 dilutions profoundly affected cell cycle, whereas higher dilutions were ineffective (data not shown). In particular, 1:120 dilution promoted an increase up to 30% of cells in sub-G0 phase, presumably dead by apoptosis, in a time-dependent manner, with a strong reduction of G0/G1 phase population, whereas 1:240 dilution induced a strong reduction of G0/G1 phase, counterbalanced by an arrest in the G2/M phase, followed by the appearance of hyperploid cells. Literature reports show that many molecules with antiproliferative effects on cancer cells, block cell cycle in the G2/M phase [46,47,48] as well as that different mechanisms could be implied, including DNA damage, or interference with formation of the mitotic spindle. Regardless of the specific mechanism involved, some of these cells can progress through a delayed mitosis and die in mitosis or finally exit mitosis, producing a single 4N G1 cell, which arrests in G1 or continues to cycle, consequently forming hyperploid cells [49]. In summary, comparing these last data with those obtained with direct cell counting, it appeared that rosemary extract could inhibit cell proliferation trough both cytotoxic and cytostatic mechanisms, in a dose and time-dependent manner, as observed for many substances with anticancer properties.

### Effect of rosemary extract treatment on intracellular ROS concentration and on protein carbonylation

**Fig 5:**
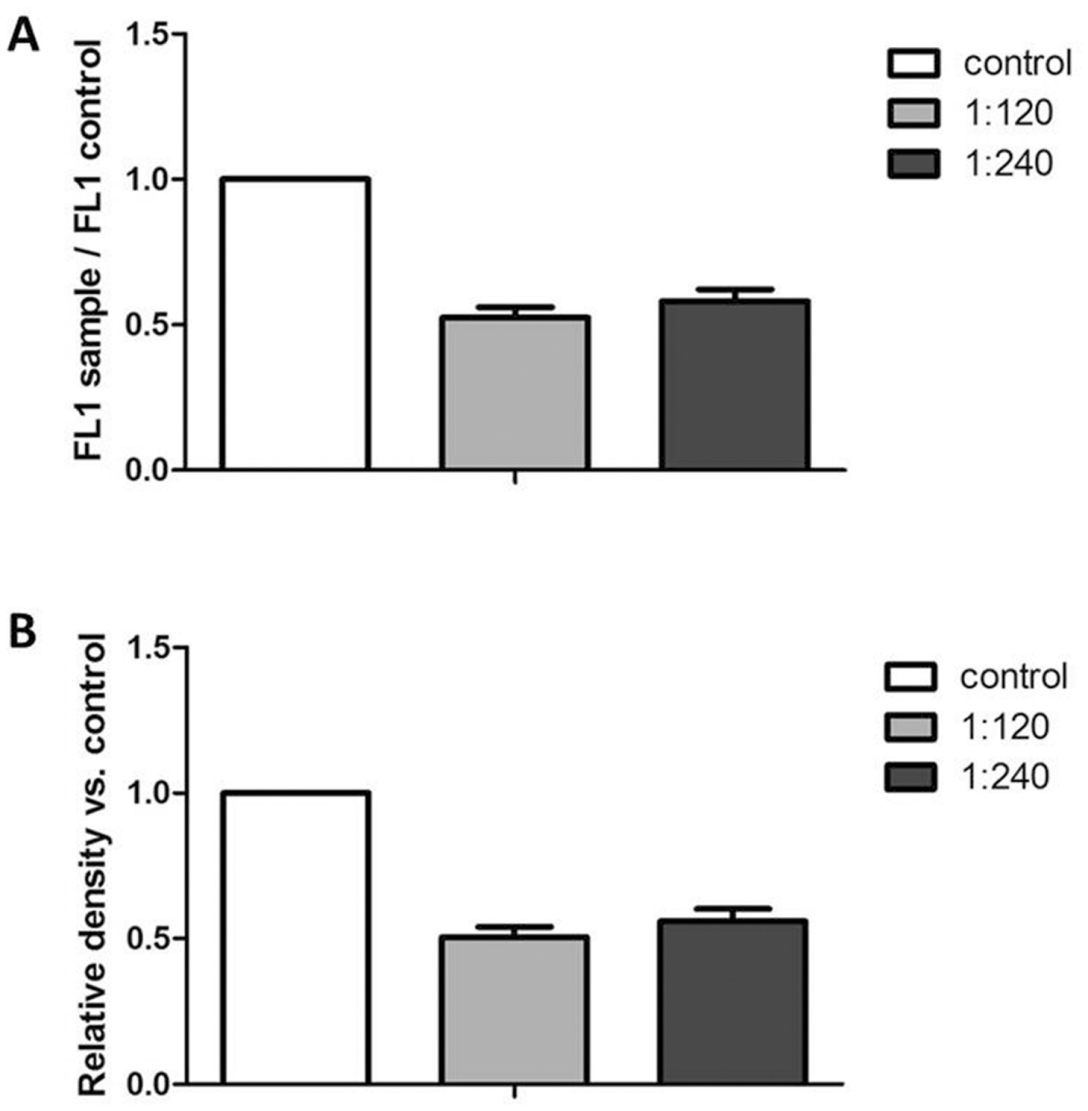
Intracellular ROS levels (panel A) and carbonylation of total proteins (panel B) of A375 melanoma cells treated with *Rosmarinus officinalis* extract. (A) Cells were incubated with 1:120 and 1:240 dilutions of the rosemary extract for 24 h. ROS production was evaluated as CM-H2DCFDA fluorescence. Values are expressed as relative fluorescence of treated samples as compared to control ones and are the mean ± SD from three independent experiments. *P≤0.05 versus vehicle treated control cells. (B) Carbonylation was evaluated by derivatization of extracted proteins with 2,4-dinitrophenylhydrazine, SDS-PAGE separation and immunoblotting with anti 2,4-dinitrophenylhydrazone antibodies. Values are expressed as relative optical density of treated samples as compared to control ones and are the mean ± SD from three independent experiments. *P≤0.05 versus vehicle treated control cells.

Since the anti-proliferative effects of different phytochemicals on various cancer cell lines has been attributed to their pro-oxidant, rather than anti-oxidant properties [50], the intracellular ROS concentration of melanoma cells treated with rosemary crude extract, compared to that of control cells, was estimated by FACS, using CM-H2DCFDA as fluorescent probe. Results from FACS analysis, reported in Fig 5 (panel A) showed that treating melanoma cells with 1: 120 and 1:240 dilutions of the extract for 24 h, brought about a significant reduction of intracellular ROS levels, thereby indicating that cytotoxicity was not triggered by cellular oxidative damage. This result was confirmed also by protein carbonylation analysis. Carbonylation is a common protein modification induced by cellular oxidative imbalance and can be easily detected by protein derivatization with DNHP and recognition with anti protein-hydrazone antibodies. The overall carbonylation level of proteins from control and treated cells was quantified by estimating the total optical density of extracted proteins after SDS PAGE, Western Blotting and immunodecoration, using the Quantity One software from Bio-rad. Results reported in Fig 5 (Panel B) demonstrated that treatments with 1:120 and 1:240 extract dilutions after 24 h incubation, determined a reduction of cell protein carbonylation, thus confirming the anti-oxidant action of the rosemary extract under our experimental conditions.

### Effect of rosemary extract treatment on the protein repertoire of melanoma cells

**Fig 6:**
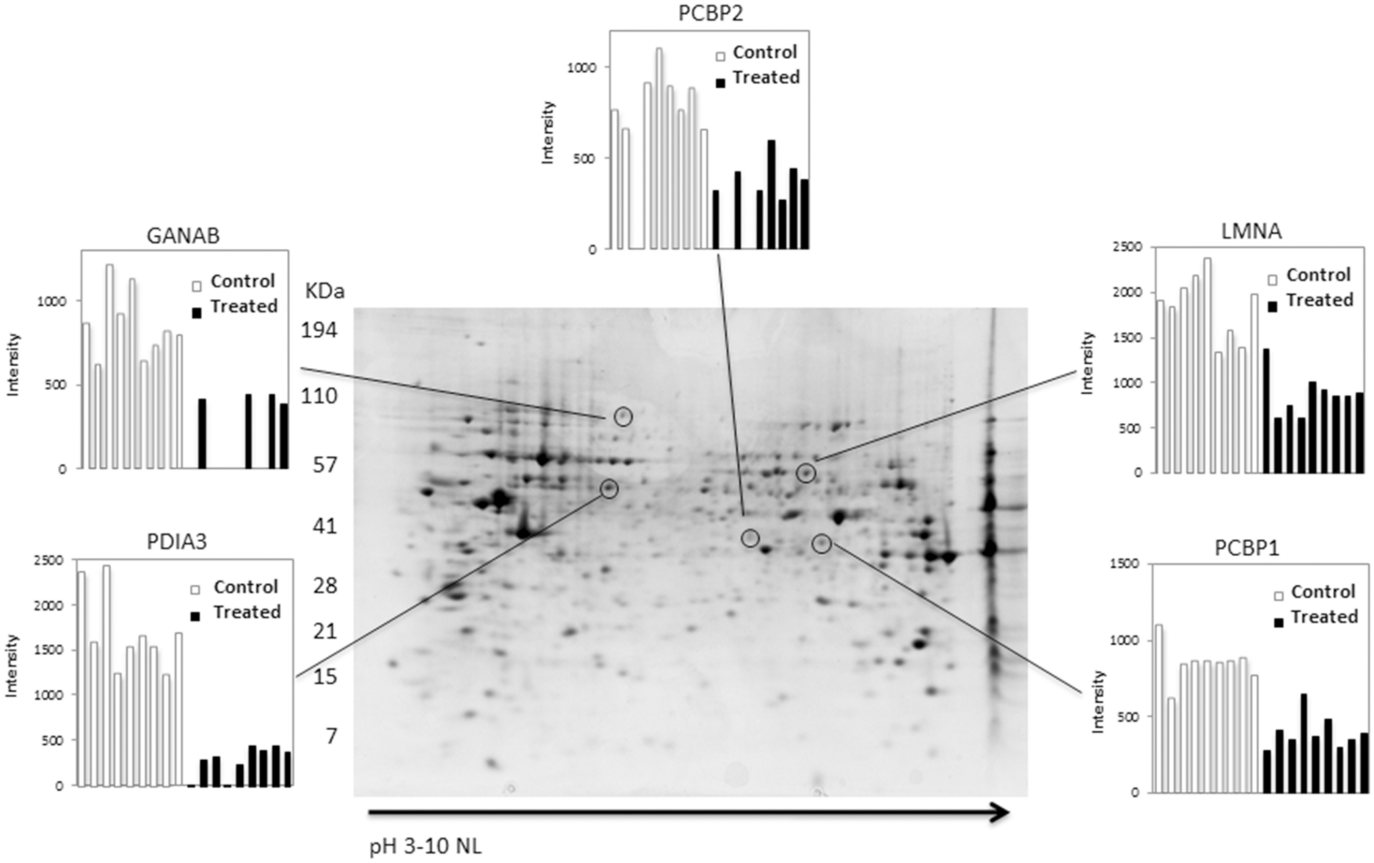
Representative 2-DE map of control A375 melanoma cells. Proteins were electrophoretically separated in the non-linear pH range 3–10 and the 200–15 kDa molecular mass range and visualized by colloidal Coomassie staining. The encircled spots indicate the proteins affected by rosemary extract (1:240; 24h) treatment. The relative intensities, between control and treated samples, are shown in panels. Data significance was evaluated by a Student’s t-test (P ≤0.05). PDA3, protein disulfide-isomerase A3; GANAB, Neutral alpha-glucosidase AB; PCBP1, poly(rC)- binding protein 1; PCBP2, poly(rC)-binding protein 2; LMNA, lamin A.

In order to get hints about the molecular mechanism underlying rosemary extract cytotoxicity, a proteomic analysis was carried out, to ascertain qualitative and/or quantitative modification of the protein profile of melanoma cells subjected to rosemary extract treatment, as compared to control cells. To this purpose, total proteins were extracted from cells treated with 1:240 dilution of rosemary extract for 24 h, or from untreated cells and resolved by two dimensional gel electrophoresis (2-DE). To detect quantitative changes in relative spot volumes of proteins from treated cells as compared to control ones, colloidal Coomassie-stained gels were subjected to software-assisted image analysis. Statistical evaluation of the relative spot volumes allowed to detect spots significantly varying (P ≤ 0.05) in abundance. Representative 2-D gel is shown in Fig 6. The overall 2-DE profiles of control and treated cells were similar, however, 5 protein spots were detected, whose abundance was at least two-fold reduced in response to rosemary challenge; no proteins with a corresponding increase were detected. The differential spots were excised from the gel, proteolysed and subjected to MS analysis. The database search with results from Peptide Mass Fingerprinting MALDI-ToF experiments, allowed the identification of protein spots. The list of the identified polypeptides is reported in Table 2. All reported proteins had good sequence coverage, significant protein scores, as well as similar experimental and theoretical Mw/pI.

**Table 2:** MALDI ToF identification of down-regulated proteins in A375 melanoma cells treated with *Rosmarinus officinalis* extract.

Protein name^a^	UniProtKB accession number	Mw/pI^b^	Mascot Score	N. of mached peptide	Sequence coverage %
Proteindisulfide-isomerase A3	P30101	57146/5.98	295	34	53
Neutralalpha-glucosidase AB	Q14697	107263/5.74	334	37	40
Lamin-A	Q5TCJ2	74380/6.57	425	63	66
Poly(rC)-bindingprotein 1	Q15365	37987/6.66	183	23	74
Poly(rC)-bindingprotein 2	Q6IPF4	38955/6.33	109	11	32

^a^Name of protein according to UniProtKb databank

^b^Theoretical molecular mass/pI

In order to verify proteomic results, validation experiments were carried out by western blotting of down-regulated proteins, probed with specific antibodies. Results, reported in S2 Fig, showed a decrease in treated samples of all of tested proteins, thereby confirming the same behaviour detected by proteomic analysis. As far as PDIA3 is concerned, the decrease observed in the western blotting analysis is less dramatic with respect to that observed in the 2-DE experiment. The reason may reside on the presence of different post-translation modifications on the PDIA3 that may alter its pI. These isoforms, dispersed in the 2DE gel, are instead all detected in the western blotting analysis.

All these identified proteins which are down-regulated could be related with the effect of rosemary treatment on melanoma cells.

Protein disulfide-isomerase A3 (PDIA3) belongs to the wide protein-disulfide isomerase family (PDI), a class of proteins which catalyzes disulfide bond formation, breakage or rearrangement and possess chaperone activity [51]. Mediating protein folding in the endoplasmic reticulum, PDIs are essential for cellular homeostasis maintenance; changes in PDI expression and/or enzymatic activity are associated with protein misfolding and ER stress at cellular level [51] and correlated to neurodegenerative and cardiovascular diseases [52]. Interestingly, a growing body of literature data indicates that PDI high expression is correlated to survival and progression of different types of cancer [52], thereby indicating that this protein could be a promising target for cancer treatment. PDIA5 has been involved in the chemoresistance mechanism mediated by activation of the oncogenic transcription factor ATF6α [53] whereas PDIA3 has been identified by a proteomic approach, as a chemoprevention target in human colon cancer cells [54]. Although the effects of PDIs in sustaining tumor progression are probably diverse, depending on the cancer type, it has been reported that, in melanoma, PDIs protected cells from apoptosis, and that their inhibition enhanced the efficacy of compounds triggering cell death [55].

Neutral alpha-glucosidase AB (GANAB), also known as glucosidase II-alpha subunit, is an ER-located enzyme which is essential to maturation of newly synthesized glycoproteins [56,57]; its disregulation causes accumulation of misfolded proteins in the ER and consequently ER stress and the unfolded protein response (UPR) [57]. Although it has been shown by a proteomic approach that its levels are strongly increased during FGF2-induced proliferation of osteoblasts [58], correlation between level and/or enzymatic activity alteration and diseases, apart from polycystic liver disease [59] is very poor. Recently, it has been shown that its down-regulation is associated to increased migration and survival of invasive lines of head and neck cancer cells [60]. At discrepancy, the observed down-regulation induced by rosemary extract treatment suggests that, at least in melanoma A375 cell line, it may be associated to reduction of cell proliferation, possibly by eliciting ER stress. Two members of the poly (rC)-binding protein family (PCB1 and PCB2) were also identified as down-regulated proteins in rosemary-treated melanoma cells. PCBs bind to single-stranded poly (C) motifs of client mRNAs and regulate diverse post-transcriptional and translational events [61]. Remarkably, different PCBs isoforms have been reported to be over-expressed in various cancers, where they appear necessary to tumor survival and development. PCB2 for instance, is up-regulated in gastric and prostate cancer cells [62,63], leukemic blasts [64] and human glioma tissue and cell lines, where knockdown of PCB2 gene inhibits glioma growth [65]. PCB1 has been identified by a proteomic study as a highly up-regulated protein in neuroendocrine pulmonary tumors [65].

Finally, a reduction of lamin A was observed as a consequence of rosemary treatment of melanoma cells. Lamins are components of the nuclear envelope, where they are essential to its organization and stability [66]. The nuclear lamina is involved in the regulation of fundamental cellular processes including DNA replication, transcription and cell cycle progression, and mutations within lamin genes give rise to a broad range of diseases known as laminopathies [66]. The link between alteration of lamin levels and cancer is poorly investigated but, interestingly, it has been shown that geraniol, inhibiting farnesylation of G proteins, among which lamin A and B, is able to suppress hepatomas and melanomas growth in transplanted rats and mice [67]. In general, factors affecting lamin maturation can lead to different downstream effects, all detrimental to cell survival and longevity, hence the observed decrease of lamin in our conditions points to a role of lamin in sustaining growth of melanoma cells, thereby suggesting that this protein may be a promising target for pharmacological therapy.

## Conclusions

In summary, this study allowed to ascertain that a 65% (v/v) hydroalcoholic extract of *Rosmarinus officinals* L. was able to efficiently reduce, in a dose and time dependent manner, the proliferation of the human melanoma A375 cell line, usually highly resistant to cytotoxic agents. Investigation of cell cycle indicated that rosemary extract inhibited cell proliferation through both cytotoxic and cytostatic effects. Evaluation of cellular ROS production and of protein carbonylation indicated that the antiproliferative effect was not due to a pro-oxidant activity of the extract. The compositional characterization carried out, allowed to test pure single components and results suggested that the antiproliferative activity was a property of the whole extract, very likely resulting from multi-factorial effects which involve most of its components. Proteomic analysis, performed in order to get hints about molecular targets involved, showed that rosemary treatment of melanoma cells induced a significant reduction of levels of proteins crucial for cellular homeostasis maintenance, whose down-regulation can hamper cellular functions by inducing ER stress. In general, *in vitro* cellular and proteomic evidence indicate that combinations of plant secondary metabolites have the potentiality to integrate chemotherapy and prompt further studies to confirm *in vivo* efficacy and to unravel the molecular basis of their polyvalent action.

## Supporting Information

S1 Fig Effect of *Rosmarinus officinalis* extract on B16-F10 murine melanoma cells. (A) Metabolic activity (MTT test). (B) Cell viability (Trypan blue exclusion test). Data are expressed as % of cell survival with respect to control. Results are the mean ± SD from three independent experiments. * P ≤ 0.05 versus vehicle control.
(TIF)

S2 Fig Expression levels of down-regulated proteins in cell after treatment with *Rosmarinus officinalis* extract. On the left, immunodetection of GANAB, LMNA, PDIA3, PCBP1 and PCBP2 in treated (+) and untreated (-) cells. All the three independent cell preparations are reported. On the right, relative intensities of the optical densities of each of the protein bands and the corresponding GAPDH band. Quantitative data are expressed as a percentage with respect to the ratio value determined in the untreated cells. Data were collected from independent cell preparations (n = 3), and averaged (%SD). Statistical analysis was performed by Student’s t-test. * P ≤ 0.05.
(TIFF)
