# Supplementary figures and images for: Anti-Proliferative Effect of Rosmarinus officinalis L. Extract on Human Melanoma A375 Cells
Source: PLoS One. 2015 Jul 15;10(7):e0132439. doi: 10.1371/journal.pone.0132439 (PMC4503536; doi:10.1371/journal.pone.0132439)

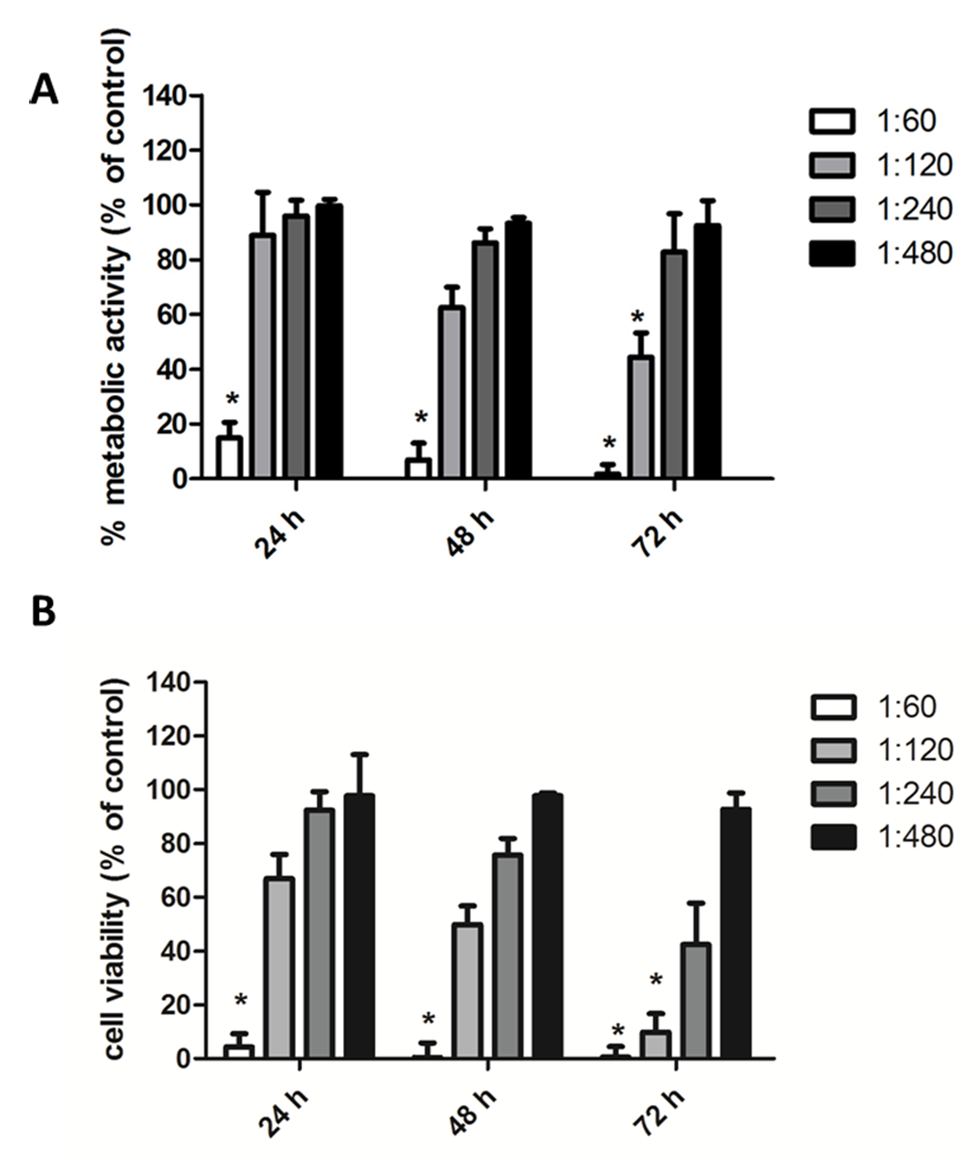

Supplement: S1 Fig — (A) Metabolic activity (MTT test). (B) Cell viability (Trypan blue exclusion test). Data are expressed as % of cell survival with respect to control. Results are the mean ± SD from three independent experiments. * P ≤ 0.05 versus vehicle control. (TIF) [file pone.0132439.s001.tif]

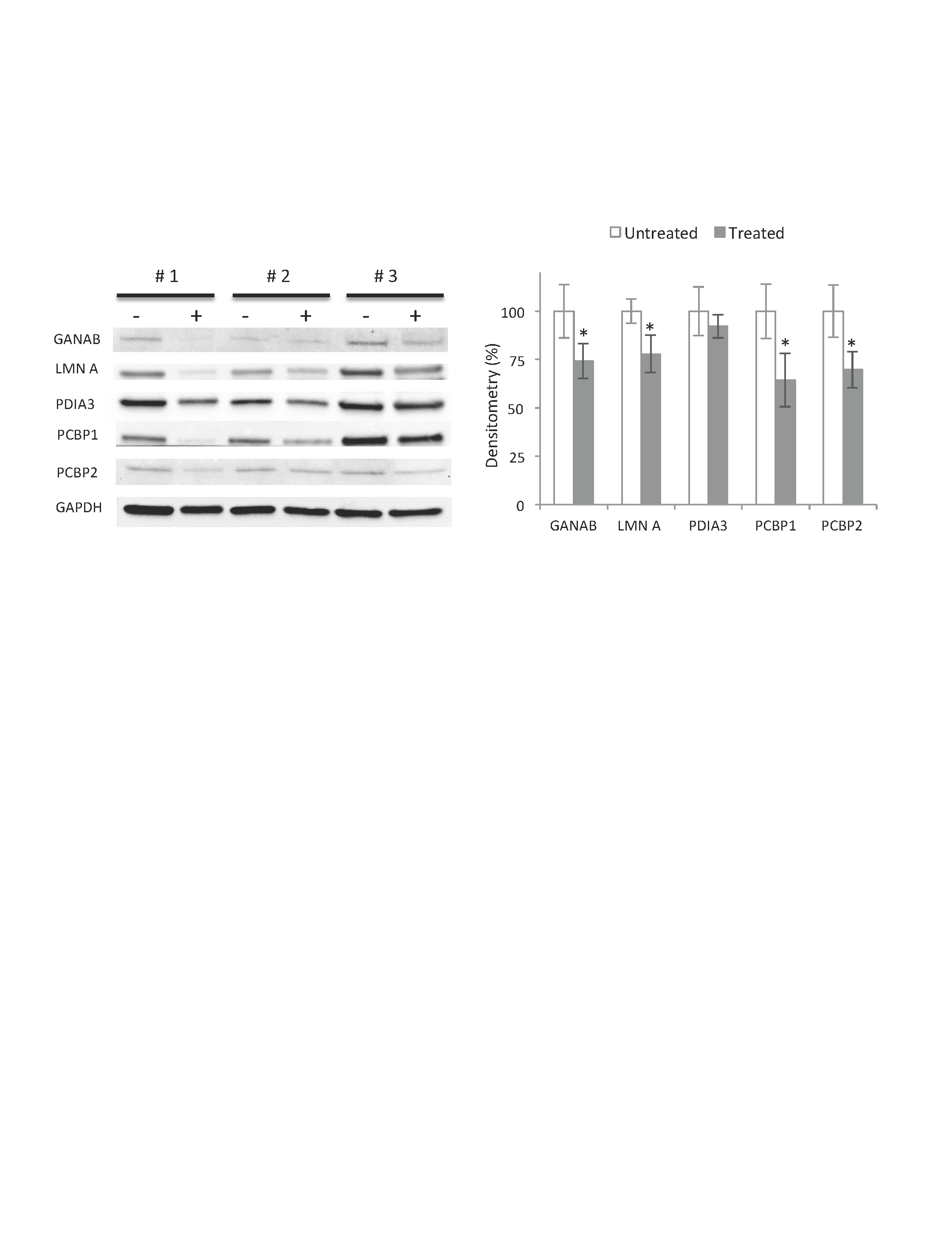

Supplement: S2 Fig — On the left, immunodetection of GANAB, LMNA, PDIA3, PCBP1 and PCBP2 in treated (+) and untreated (-) cells. All the three independent cell preparations are reported. On the right, relative intensities of the optical densities of each of the protein bands and the corresponding GAPDH band. Quantitative data are expressed as a percentage with respect to the ratio value determined in the untreated cells. Data were collected from independent cell preparations (n = 3), and averaged (%SD). Statistical analysis was performed by Student’s t-test. * P ≤ 0.05. (TIFF) [file pone.0132439.s002.tiff]
